# Convergent and Divergent Structural Connectivity of Brain White Matter Network Between Patients With Erectile Dysfunction and Premature Ejaculation: A Graph Theory Analysis Study

**DOI:** 10.3389/fneur.2022.804207

**Published:** 2022-02-22

**Authors:** Tielong Zhang, Peng Yuan, Yonghua Cui, Weibiao Yuan, Daye Jiang

**Affiliations:** ^1^Department of Urology, The Affiliated Jianhu Hospital of Nantong University, Jianhu People's Hospital, Yancheng, China; ^2^Department of Intervention, The Affiliated Jianhu Hospital of Nantong University, Jianhu People's Hospital, Yancheng, China; ^3^Department of Neurosurgery, The Affiliated Jianhu Hospital of Nantong University, Jianhu People's Hospital, Yancheng, China; ^4^Department of Radiology, The Affiliated Jianhu Hospital of Nantong University, Jianhu People's Hospital, Yancheng, China

**Keywords:** erectile dysfunction, premature ejaculation, diffusion tensor imaging, graph theory analysis, structural connectivity

## Abstract

**Background:**

Sexual dysfunction, namely, erectile dysfunction (ED) and premature ejaculation (PE), has been found to be associated with abnormal structural connectivity in the brain. Previous studies have mainly focused on a single disorder, however, convergent and divergent structural connectivity patterns of the brain network between ED and PE remain poorly understood.

**Methods:**

T1-weighted structural data and diffusion tensor imaging data of 28 patients with psychological ED, 28 patients with lifelong PE (LPE), and 28 healthy controls (HCs) were obtained to map the white matter (WM) brain networks. Then, the graph-theoretical method was applied to investigate the differences of network properties (small-world measures) of the WM network between patients with ED and LPE. Furthermore, nodal segregative and integrative parameters (nodal clustering coefficient and characteristic path length) were also explored between these patients.

**Results:**

Small-world architecture of the brain networks were identified for both psychological ED and LPE groups. However, patients with ED exhibited increased average characteristic path length of the brain network when compared with patients with LPE and HCs. No significant difference was found in the average characteristic path length between patients with LPE and HCs. Moreover, increased nodal characteristic path length was found in the right middle frontal gyrus (orbital part) of patients with ED and LPE when compared with HCs. In addition, patients with ED had increased nodal characteristic path length in the right middle frontal gyrus (orbital part) when compared with patients with LPE.

**Conclusion:**

Together, our results demonstrated that decreased integration of the right middle frontal gyrus (orbital part) might be a convergent neuropathological basis for both psychological ED and LPE. In addition, patients with ED also exhibited decreased integration in the whole WM brain network, which was not found in patients with LPE. Therefore, altered integration of the whole brain network might be the divergent structural connectivity patterns for psychological ED and LPE.

## Introduction

Male sexual behavior is divided into five stages: sexual desire, sexual arousal/erection, sexual intercourse, ejaculation, and orgasm (1, 2). In humans, penile erection and ejaculation can occur during masturbation, copulation, or sleep, even in some non-sexually relevant context (3, 4). It is possible that certain brain regions may contribute to the occurrence of erection and ejaculation in different contexts (5). Previous studies have demonstrated that erection and ejaculation are considered two distinct phases of male sexual behavior, which are controlled by the peripheral and central nervous systems (6–9). Erectile dysfunction (ED) and premature ejaculation (PE) are the two most common sexual dysfunctions with many little-known links, which have a negative impact on the physical and psychosocial health and quality of sexual intercourse for both men and their female partners (10, 11). Approximately, 5–20% of men suffer from moderate-to-severe ED including psychological ED (12) and 20–30% of men report experiencing PE including lifelong PE (LPE) at some point in their lives (13, 14). However, the diagnosis of both the psychological ED and LPE is usually based on medical and sexual history and validated questionnaires assessing the level of ED and PE (15).

The links between certain brain areas and erection have been indirectly evidenced by the proerectile effects of apomorphine in the treatment of some patients suffering from cerebral dopamine deficiency (16, 17). Furthermore, more attention was paid to understanding how brain regions modulate the process of ejaculation with the use of selective serotonin reuptake inhibitors in delaying ejaculation (18). The central neuropathological mechanisms of male sexual dysfunction recently received significant attention with the development of MRI, a non-invasive imaging technique, for displaying the structure and function of the brain (5, 19). Diffusion tensor imaging (DTI), one of the most used neuroimaging methods, can identify structural connections and explore changes in the white matter (WM) microstructure *in vivo* (20). Graph theory, a mathematical analysis method, quantifies the whole brain as a graph consisting of nodes linked by edges (21, 22). Graph analysis has revealed that the human brain network exhibits small-world network patterns, which have a balance between high segregation (measured by clustering coefficient) and integration (measured by characteristic path length) (23, 24). The function of the prefrontal cortex in emotion, cognition, and motivation has been mostly studied in domains other than sexual behavior (25). The role of the prefrontal cortex, especially the lateral prefrontal cortex, may be associated with the sexual arousal in response to sexual stimuli and may be involved in processing the sexual character of stimuli (26, 27). In addition, the activation of the orbitofrontal cortex, one component of the central inhibitory network, may be involved in the central control of ejaculation (28). Both patients with psychological ED (29) and LPE (30) showed impaired functional and structural connectivity in the brain in previous MRI studies. The altered brain regions were mainly located in the frontal cortex including the orbital prefrontal region and subcortical areas, especially the amygdala. However, convergent and divergent brain mechanisms underlying psychological ED and LPE remain poorly understood.

In this study, we used DTI data and the graph theory approach to investigate the convergent and divergent structural connectivity patterns of the brain network between patients with psychological ED and LPE. Based on previous studies, we hypothesized that psychological ED and LPE-related differences (mainly in the prefrontal cortex) would occur in the patterns of structural connectivity in the brain. We sought to determine whether WM networks would show (1) abnormal topological organization (small-world patterns) in patients with psychological ED and LPE; (2) similarities and differences in the patterns of structural connectivity (nodal clustering coefficient and characteristic path length of the prefrontal cortex) between these two groups.

## Materials and Methods

### Participants

A total of 28 patients with psychological ED and 28 patients with LPE were recruited from the Department of Urology, the Affiliated Jianhu Hospital of Nantong University, Jianhu People's Hospital. In addition, 28 well-matched healthy controls (HCs) were recruited by advertisement. All the participants underwent medical and sexual history taking and physical examination.

Diagnostic criteria: (1) ED: patients with ED met the Diagnostic and Statistical Manual of Mental Disorders-Fifth Edition (DSM-V) (31) criteria (failure to obtain and maintain an erection sufficient for sexual activity or decreased erectile turgidity on 75% of sexual occasions and lasting for at least 6 months), (2) LPE: patients with LPE met the International Society for Sexual Medicine (ISSM) criteria (32) for LPE [(i) a short ejaculatory latency; (ii) a lack of perceived self-efficacy or control about the timing of ejaculation; and (iii) distress and interpersonal difficulty (related to the ejaculatory dysfunction)].

Evaluation tools: (1) psychological ED: scores of international index of erectile function (IIEF-5) (33, 34) ≤21, scores of premature ejaculation diagnostic tool (PEDT) (35, 36) <11, normal nocturnal erection and normal erection during masturbation reported by themselves. (2) LPE: PEDT scores ≥ 11, IIEF-5 scores > 21, and intravaginal ejaculation latency time (IELT) < 1 min since the first experience of sexual intercourse.

The inclusion criteria for HCs were a total score of IIEF-5 > 21, PEDT < 11, and IELT > 3 min. The inclusion criteria for all participants: (1) right-handed; (2) aged from 20 to 45 years old; (3) drug washout period (any treatment influences sexual function) >2 weeks; (4) frequency of sexual activity >4 times/week; (5) in a stable relationship with the same, non-pregnant, sexually active female partner for at least 6 months. Exclusion criteria for all participants were: (1) genital deformities including short frenulum evaluated by physical examination and other sexual dysfunction, such as hypoactive sexual desire disorder, anejaculation, and retrograde ejaculation; (2) head trauma, loss of consciousness, psychiatric diseases including depression, and anxiety evaluated by self-rating depression and anxiety scales/neurological diseases and other serious physical diseases, namely, hypertension, diabetes, coronary heart disease, liver and kidney diseases, tumor, etc.; (3) alcohol or drug abuse; and (4) any contraindications for MRI scanning.

Our study was approved by the Ethical Committee of the Affiliated Jianhu Hospital of Nantong University, Jianhu People's Hospital. Written informed consent was obtained from all participants before the study. The demographic and clinical information for all participants were presented in Table 1.

**Table 1:** Demographic and clinical data.

**Variables**	**Psychological ED (*n* = 28)**	**LPE (*n* = 28)**	**HCs (*n* = 28)**	***P***
Age (years)	32.82 ± 2.23	30.29 ± 4.13	31.39 ± 6.72	0.13
Education level (years)	14.86 ± 1.96	14.54 ± 2.62	14.57 ± 1.60	0.40
IIEF-5 scores	10.86 ± 3.66	22.61 ± 0.69	22.71 ± 0.76	0.000015^a^
PEDT scores	3.96 ± 2.01	14.64 ± 3.53	3.68 ± 1.79	<0.0001^b^
IELT (seconds)	366.43 ± 117.51	30.68 ± 18.56	405.00 ± 98.83	<0.0001^c^

*ED, erectile dysfunction; LPE, lifelong premature ejaculation; HCs, healthy controls; IELT, intravaginal ejaculation latency time; PEDT, premature ejaculation diagnostic tool*.

a *Significant differences were found in psychological ED when compared with LPE and HCs*.

b, c *Significant differences were found in LPE when compared with psychological ED and HCs. Multiple and two independent samples non-parametric tests were conducted to explore the differences of demographic and clinical data among the three groups. P < 0.05 was considered as statistically significant*.

### Magnetic Resonance Imaging Data Acquisition and Processing

Magnetic resonance imaging data were acquired using a 3.0 Tesla GE scanner (GE Company, America). T1-weighted images were acquired with the following parameters: repetition time (TR) = 8.5 ms, echo time (TE) = 3.2 ms, matrix size = 256 × 256, and slice thickness = 1 mm. DTI data were acquired with the following parameters: TR = 8,724 ms, TE = 81.4 ms, slice thickness = 2 mm, 64 diffusion directions with b = 1,000 s/mm^2^, and an additional b0 image. Participants were instructed to close their eyes and stay awake during the MRI data acquisition.

MRI data processing was implemented using the FMRIB Diffusion Toolbox (FDT) in FMRIB's Software Library (37). The data processing procedure was as follows: (1) brain extraction; (2) corrections for head motion artifacts and eddy current distortions in the DTI dataset by registering the diffusion-weighted images to the b0 images; (3) diffusion tensor estimation by the Stejskal and Tanner equation (38); (4) calculation of fractional anisotropy (FA) (the extent of directionality of water diffusion can be expressed as FA, which is a measure reflecting the directional organization of WM in the brain) of each voxel and FA maps describing the distribution of WM fiber tracts in the whole brain; (5) reconstruction of WM pathways using fiber assignment by continuous tracking (FACT) algorithm.

In this study, the parcellation process was performed in the native space. First, the T1-weighted images of each subject were co-registered to their corresponding b0 images in the DTI space. Second, the coregistered T1-weighted images were normalized to the International Consortium of Brain Mapping 152 T1 template in the Montreal Neurological Institute (MNI) space. Lastly, the inverse transformations were applied to warp the automated anatomical labeling template from the MNI space to the DTI native space (39). In addition, the tractography was terminated if the turned an angle >50° or FA value of a reached voxel <0.2.

### Brain Network Construction and Analysis

The WM brain networks were constructed as described in previous studies (40, 41). Nodes (brain regions) and edges (structural connectivity/WM fiber tracts) are the two fundamental elements of a network (structural/WM brain network) (42). To define the nodes, the entire cerebral cortex was divided into 90 cortical and subcortical regions (45 regions in each hemisphere). To define the edges, a threshold of 3 fiber streamlines was selected and the mean FA value of the fiber connected two brain regions was defined as the weight of the edge between these regions. Finally, FA-weighted 90 × 90 WM brain networks of all the subjects were constructed.

The small-world measures include the clustering coefficient (*C*_*p*_) (a measure of segregation; local information transformation capacity), characteristic path length (*L*_*p*_) (a measure of integration; global information transformation capacity) of the network, their corresponding normalized *C*_*p*_ (γ) and *L*_*p*_ (λ), and small-worldness (σ) (the balance between local segregation and global integration) (42). These measures were calculated to evaluate the information integration and segregation of the whole brain network using the Brain Connectivity Toolbox. In addition, the nodal parameters, namely, nodal clustering coefficient (*C*_*i*_) and characteristic path length (*L*_*i*_) were calculated to explore the integration and segregation of the brain regions in the network.

### Statistical Analysis

Statistical analyses were performed by IBM SPSS statistics (version 20) for Windows (IBM Corporation, Armonk, New York, USA). Multiple and two independent samples non-parametric tests were conducted to explore the differences of demographic and clinical data among the three groups (*P* < 0.05). Then *ANOVA* was employed to identify group differences in the graph metrics of both network (*P* < 0.05) and node (*P* < 0.01). In addition, false discovery rate (FDR) correction (number of tests = 90 (brain regions); *P*_*i*_ < 0.05 × *i*/90; *P*_1_ ≤ *P*_2_ ≤ *P*_3_… ≤ *P*_*i*_; *i* = 1, 2, 3…90) was applied to account for multiple comparisons of nodal parameters calculations.

## Results

### Global Network Properties

**Figure 1:**
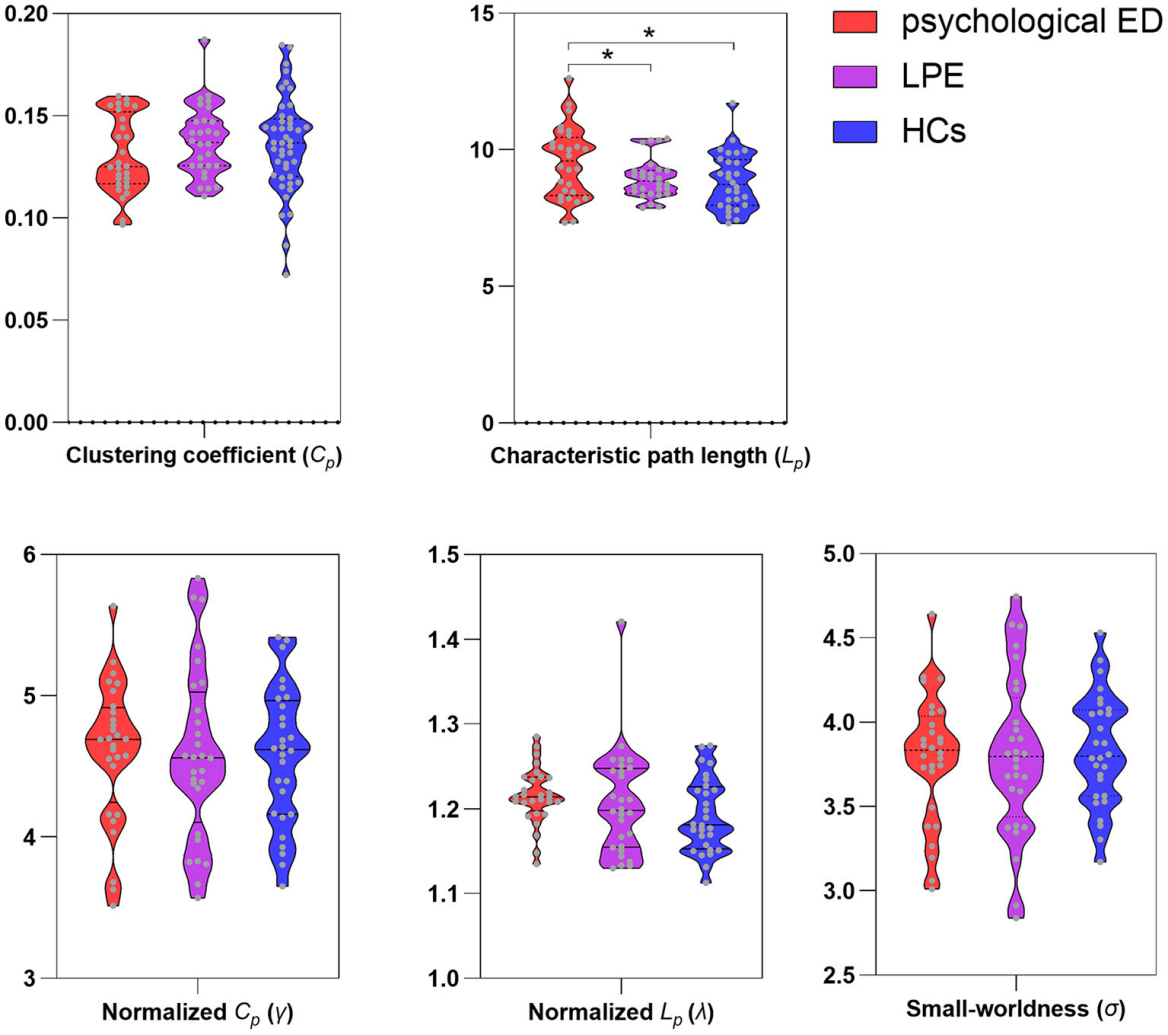
Differences of small-world measures among three groups. ED, erectile dysfunction; LPE, lifelong premature ejaculation; HCs, healthy controls. *Significant differences between two groups detected by two-sample *t*-test. *P* < 0.05 was considered as statistically significant.

Small-world properties were demonstrated in both the patients and HCs (γ >> 1; λ ≈ 1; σ >> 1). However, patients with psychological ED, relative to patients with LPE and HCs, showed significant increases in the average characteristic path length *L*_*p*_ of the brain network. No significant differences were found in the average characteristic path length *L*_*p*_ of the brain network between patients with LPE and HCs. Concerning other small-world measures (*C*_*p*_; γ; λ; σ), no significant differences were found among the three groups (Figure 1 and Table 2).

**Table 2:** Differences of small-world measures in the white matter brain networks.

**Network metrics**	**Psychological ED (*n* = 28)**	**LPE (*n* = 28)**	**HCs (*n* = 28)**	***F***	***P***
Clustering coefficient (*C_*p*_*)	0.13 ± 0.019	0.14 ± 0.018	0.14 ± 0.020	1.29	0.28
Characteristic path length (*L_*p*_*)	9.54 ± 1.32	8.95 ± 0.72	8.85 ± 1.05	3.51	0.035^a^
Normalized *C_*p*_* (γ)	4.61 ± 0.50	4.59 ± 0.61	4.57 ± 0.49	0.027	0.97
Normalized *L_*p*_* (λ)	1.22 ± 0.034	1.21 ± 0.062	1.19 ± 0.044	1.62	0.20
Small-worldness (σ)	3.79 ± 0.38	3.81 ± 0.48	3.83 ± 0.33	0.079	0.92

*ED, erectile dysfunction; LPE, lifelong premature ejaculation; HCs, healthy controls*.

a *Significant differences were found in psychological ED when compared with LPE and HCs. One-way ANOVA was performed among three groups and two-sample t-test was performed as post-hoc tests. P < 0.05 was considered as statistically significant*.

### Regional Nodal Characteristics

**Figure 2:**
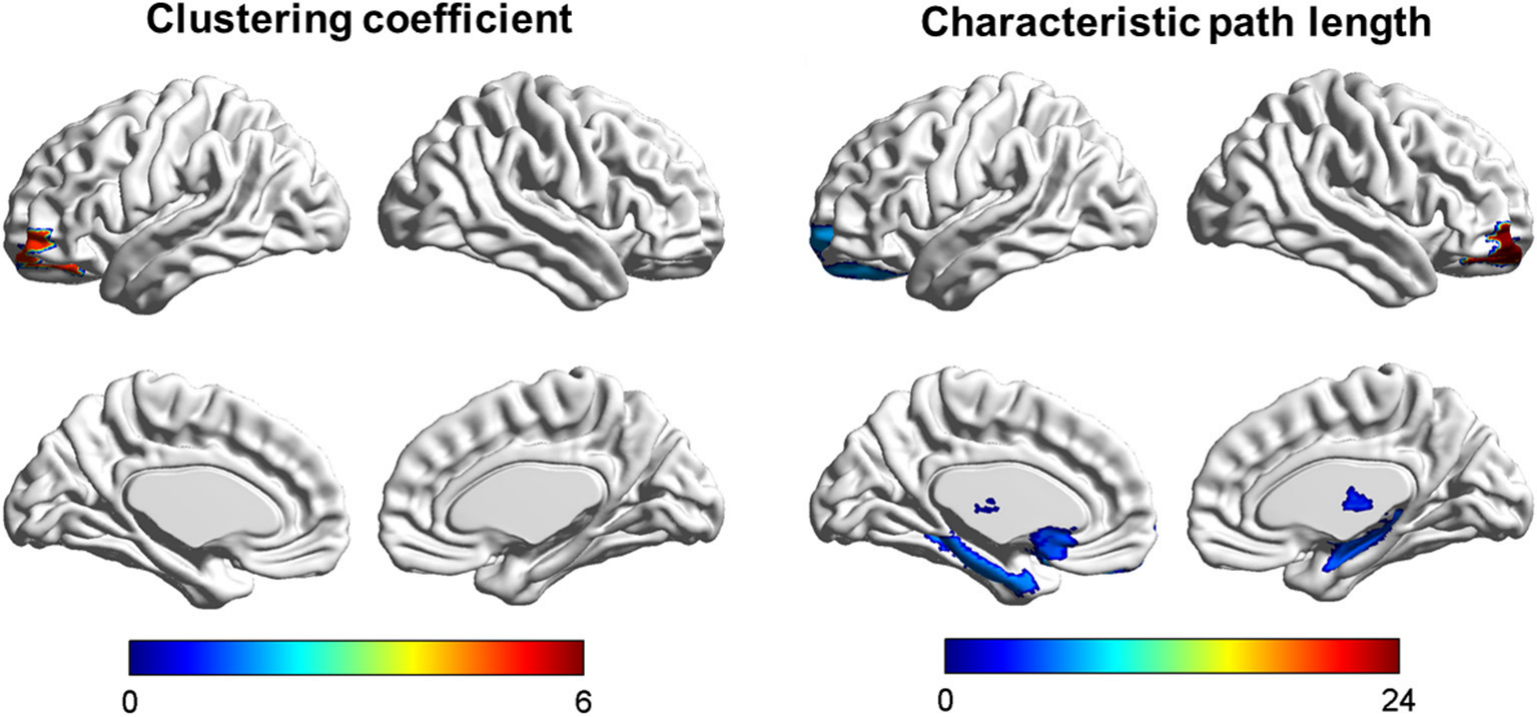
Brain areas showing abnormal segregation and integration among the three groups. Color bar: *F*-values detected by one-way *ANOVA* test. *P* < 0.01 was considered as statistically significant.

**Figure 3:**
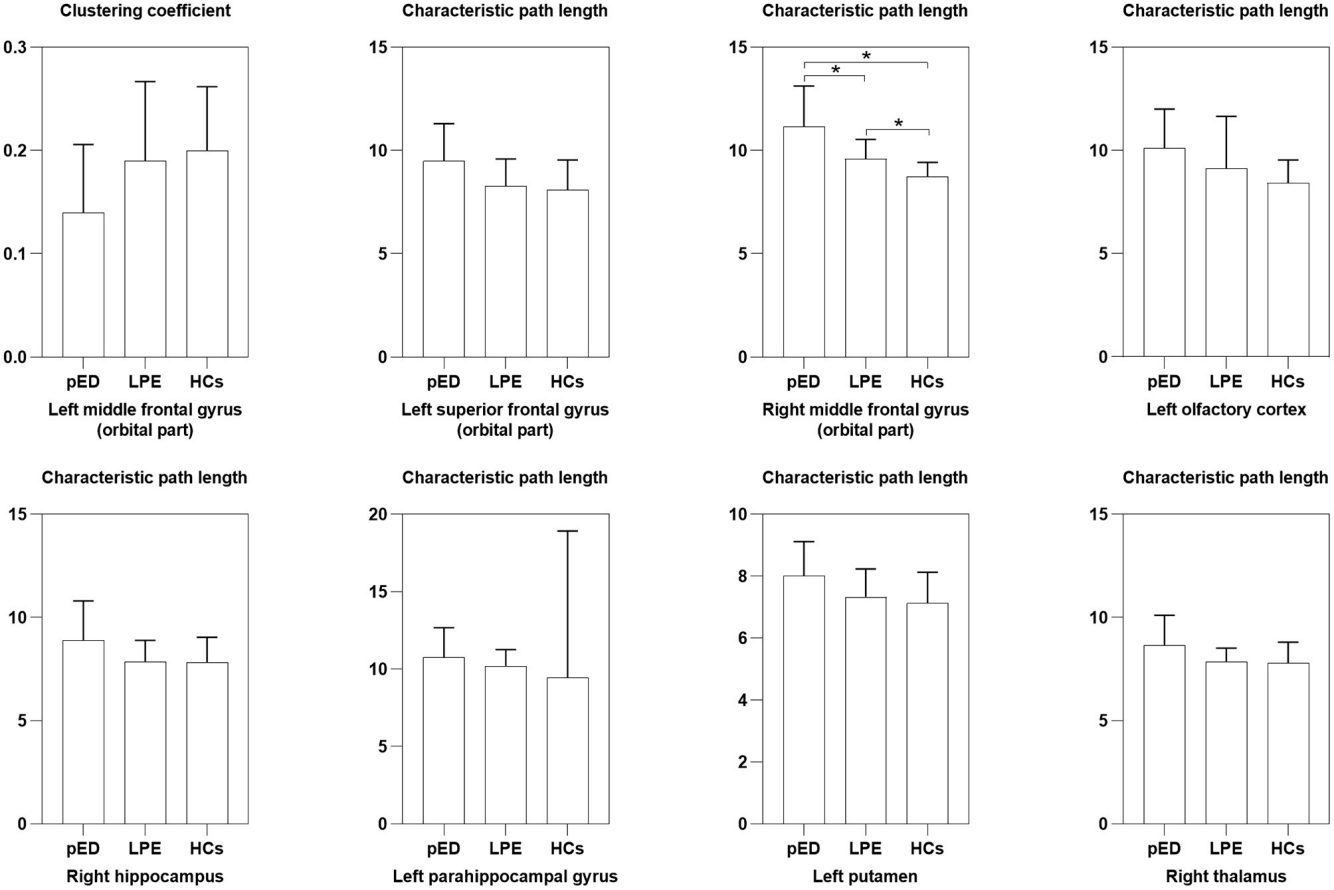
Differences of nodal segregative and integrative parameters among the three groups. pED, psychological erectile dysfunction; LPE, lifelong premature ejaculation; HCs, healthy controls. *Significant differences between two groups detected by two-sample *t*-test. *P* < 0.01 was considered as statistically significant.

Among the three groups, we found that the regions with significant group effects of nodal characteristic path length (*L*_*i*_) were mainly distributed in the frontal and subcortical regions (Figure 2, Table 3). However, only the right middle frontal gyrus (orbital part) showed significant group differences after FDR correction (Figure 3, Table 3). Compared to HCs, both patients with psychological ED and LPE exhibited increased nodal characteristic path length (*L*_*i*_) in the right middle frontal gyrus (orbital part). Moreover, patients with psychological ED showed increased nodal characteristic path length in the right middle frontal gyrus (orbital part) when compared with patients with LPE.

**Table 3:** Differences of nodal segregative and integrative parameters in the white matter brain networks.

**Nodal metrics**	**Psychological ED (*n* = 28)**	**LPE (*n* = 28)**	**HCs (*n* = 28)**	***F***	***P***
**Nodal clustering coefficient (** ***C**_***i***_* **)**					
Left middle frontal gyrus (orbital part)	0.14 ± 0.066	0.19 ± 0.077	0.20 ± 0.062	5.47	0.0059
**Nodal characteristic path length (** ***L**_***i***_* **)**					
Left superior frontal gyrus (orbital part)	9.49 ± 1.81	8.28 ± 1.32	8.11 ± 1.44	6.67	0.0021
Right middle frontal gyrus (orbital part)	11.15 ± 1.98	9.60 ± 0.94	8.73 ± 0.70	23.73	<0.0001^a^
Left olfactory cortex	10.12 ± 1.89	9.14 ± 2.52	8.43 ± 1.11	5.47	0.0059
Right hippocampus	8.89 ± 1.92	7.87 ± 1.02	7.82 ± 1.22	4.94	0.0094
Left parahippocampal gyrus	10.77 ± 1.90	10.20 ± 1.06	9.46 ± 1.33	5.63	0.0051
Left putamen	8.01 ± 1.11	7.33 ± 0.91	7.13 ± 1.00	5.79	0.0044
Right thalamus	8.66 ± 1.45	7.87 ± 0.65	7.78 ± 1.03	5.48	0.0059

*ED, erectile dysfunction; LPE, lifelong premature ejaculation; HCs, healthy controls*.

a *Significant differences were found between all groups. One-way ANOVA was performed among the three groups and two-sample t-test was performed as post-hoc tests. P < 0.01 was considered as statistically significant*.

## Discussion

In this study, we evaluated the architecture of WM networks in patients with ED (psychological ED characterized by normal nocturnal erection and normal erection during masturbation) and LPE, which presented similar small-world organization. However, patients with psychological ED exhibited increased characteristic path length *L*_*p*_ when compared with patients with LPE. Moreover, significantly increased nodal characteristic path length (*L*_*i*_) was found in patients with psychological ED and LPE compared with HCs, mainly located in the right middle frontal gyrus (orbital part). For patients with psychological ED, a significant decline of *L*_*i*_ was found in the right middle frontal gyrus (orbital part) when compared with patients with LPE. These findings improved our understanding of convergent and divergent neuropathological mechanisms in the brain WM network level between psychological ED and LPE.

Previous studies showed that ED and PE were strongly correlated with psychological factors (e.g., alexithymia) and these patients were lack of emotional awareness and shared the same difficulty in identifying and communicating emotions (i.e., alexithymia) (43–45). The brain functional and structural imaging results demonstrated that the development of alexithymia might be caused by the altered connectivity among the superior frontal gyrus, inferior temporal gyrus, anterior cingulate gyrus, and insula (46, 47). In addition, an increasing body of neuroimaging evidence has identified structural brain abnormalities in psychogenic and venous ED, and LPE, mainly in the prefrontal cortex, limbic system, and subcortical areas (48–51). Previous studies have revealed impaired WM in the prefrontal cortex of patients with psychological ED and LPE (52, 53). In addition, ED owing to psychological factors and patients with PE having depressive symptoms had altered topological characteristics in the prefrontal regions of the WM brain network (29, 54). Regarding the global topological organization, the small world is one of the major organizational principles of the human brain (24). We found a conserved small-world architecture in both patients with psychological ED and LPE, and in HCs, which was consistent with previous studies (52, 55). This demonstrated that the human brain had evolved into a complex but efficient interconnected system, which was capable of parallel information processing with high efficiency at a low cost (24). However, patients with psychological ED exhibited significantly increased characteristic path length *L*_*p*_ of the brain network when compared with both patients with LPE and HCs. The average characteristic path length *L*_*p*_ of the brain network represents the averaged ability of information transformation between regions in the whole brain. Therefore, this finding of increased *L*_*p*_ might suggest that the information transfer between brain regions became more difficult with higher wiring cost (the lower ability of the brain to globally integrate information) in patients with psychological ED.

Regarding the nodal topological properties, significant increased nodal characteristic path length (*L*_*i*_) was discovered in both patients with psychological ED and LPE, mainly located in the right middle frontal gyrus (orbital part). The measure of *L*_*i*_ represents the ability of information transfer from one region to other regions in the whole brain (42). Increased *L*_*i*_ reflected the disruption of brain regions' structural connection, which suggested decreased parallel information transfer between distant brain regions. The prefrontal cortex is the most complex and highly evolved neocortex region, which accepts different afferent nerve fibers from other brain areas (56, 57). The prefrontal cortex was thought to play a central role in the pathophysiology of male sexual dysfunctions in previous functional and structural MRI studies (5, 58, 59).

The middle frontal gyrus has been found to be involved functionally in cognition and emotional regulation (60, 61). The middle frontal gyrus (orbital part), a region of the orbitofrontal cortex is implicated with the inhibitory control of the human brain (62, 63). Previous studies demonstrated that the process of ejaculation was associated with decreased activity in this area (64–66). The orbitofrontal cortex was thought to modulate the activity of the amygdala, which showed increased activity in patients with PE (48, 67). The PE-related abnormalities of function and structure were observed in the frontal regions, namely, the orbital frontal cortex, superior frontal gyrus (medial), middle frontal gyrus (orbital part), inferior frontal gyrus (orbital part), and cingulate gyrus (49, 59, 64, 65, 68). These findings suggested that patients with PE had an abnormal brain control network, which might contribute to the reduced central control of rapid ejaculation (28). Therefore, decreased ability of information integration in the middle frontal gyrus (orbital part) might contribute to the reduced inhibition of ejaculatory reflex in patients with PE. The increased nodal characteristic path length (*L*_*i*_) of the right middle frontal gyrus (orbital part) might be involved in the psychopathology and pathophysiology of rapid ejaculation in patients with LPE.

The middle frontal gyrus is also an important part of the lateral prefrontal cortex, which has been found to be actively involved in maintaining the representation of sexual information in working memory (5, 27). Previous studies showed that the lateral prefrontal cortex had a critical role in male sexual arousal and sexual behavior (27, 69). The middle frontal gyrus (orbital part) is a core region of the central executive network in the brain, which is primarily implicated in cognitive control, response inhibition, and attention (60, 70). The previous study demonstrated that patients with minor changes (fewer subregions) of the lateral prefrontal cortex exhibited inhibition in behavior while patients with superior abnormalities (more subregions) of this region had both aberrant sexual inhibition and insufficient cognition and attention to sexual targets (27, 71). This finding illustrated that superior increased nodal characteristic path length (*L*_*i*_) of the right middle frontal gyrus (orbital part) might be an important neural pathological feature of psychological ED, which was different from PE. Therefore, we speculated that the WM brain network of psychological ED was more sensitive than that of patients with LPE and psychological ED had severely impaired integration in the brain than LPE. Both ED and PE, especially psychological ED and LPE might be related to impaired structural brain connectivity. For these patients, more targeted treatments to improve brain functional and structural connectivity should be considered.

There were several limitations in this study. First, this was a cross-sectional study, which could not find the causal relationships between abnormal measures of WM network and male sexual dysfunction. Second, the sample size of this study was relatively small and future longitudinal studies with large sample sizes were of particular interest in this regard. Third, the lack of standardized sexual evaluation was another limitation, especially the methods used to distinguish psychological sexual dysfunction and organic sexual dysfunction. Finally, patients with psychological ED included in this study were considered to be suffered from psychogenic factors, which might be also related to the altered brain connectivity. Therefore, the relationships between psychological ED and psychogenic factors should be further explored.

## Conclusion

In conclusion, the results of this study demonstrated that the brain WM network of both patients with psychological ED and LPE had small-world organization. However, decreased global integration of the brain was found in patients with psychological ED, which was the divergent structural connectivity patterns in the brain network level between psychological ED and LPE. Moreover, the right middle frontal gyrus (orbital part) exhibited more remarkably decreased integration in patients with psychological ED when compared with patients with LPE. This study provided another perspective for understanding the convergent and divergent neuropathological mechanisms between psychological ED and LPE.

## Data Availability Statement

The raw data supporting the conclusions of this article will be made available by the authors, without undue reservation.

## Ethics Statement

The studies involving human participants were reviewed and approved by the Ethical Committee of the Affiliated Jianhu Hospital of Nantong University, Jianhu People's Hospital. The patients/participants provided their written informed consent to participate in this study.

## Author Contributions

TZ and DJ designed the experiments, wrote the manuscript, and approved the final manuscript. TZ, DJ, PY, and WY contributed to clinical data collection and assessment. TZ, DJ, and YC analyzed the results. All authors contributed to the article and approved the submitted version.

## Funding

This study was supported by the grants of Yancheng Medical Technology Development Project (No. YK2015076), 333 Project of Jiangsu Province (No. BRA2017219), and Medical project of Jiangsu Health Commission (No. 233).

## Conflict of Interest

The authors declare that the research was conducted in the absence of any commercial or financial relationships that could be construed as a potential conflict of interest.

## Publisher's Note

All claims expressed in this article are solely those of the authors and do not necessarily represent those of their affiliated organizations, or those of the publisher, the editors and the reviewers. Any product that may be evaluated in this article, or claim that may be made by its manufacturer, is not guaranteed or endorsed by the publisher.
